# Quantification of NADH:ubiquinone oxidoreductase (complex I) content in biological samples

**DOI:** 10.1016/j.jbc.2021.101204

**Published:** 2021-09-17

**Authors:** Fariha Ansari, Belem Yoval-Sánchez, Zoya Niatsetskaya, Sergey Sosunov, Anna Stepanova, Christian Garcia, Edward Owusu-Ansah, Vadim Ten, Ilka Wittig, Alexander Galkin

**Affiliations:** 1 Division of Neonatology, Department of Pediatrics, Columbia University Medical Center, New York, New York, USA; 2 Department of Physiology & Cellular Biophysics, Columbia University, New York, New York, USA; 3 Functional Proteomics, Institute of Cardiovascular Physiology, Goethe University, Frankfurt am Main, Germany; 4 German Center for Cardiovascular Research (DZHK), Partner site RheinMain, Frankfurt am Main, Germany; 5 Feil Family Brain and Mind Research Institute, Weill Cornell Medicine, New York, New York, USA

**Keywords:** mitochondrial respiratory chain complex I, flavin mononucleotide, flavin adenine dinucleotide, fluorescence, stoichiometry, enzyme turnover, ketoglutarate dehydrogenase complex, BSA, bovine serum albumin, DDM, *n*-dodecyl-β-d-maltoside, DLD, dihydrolipoyl dehydrogenase, FAD, flavin adenine dinucleotide, HAR, hexaammineruthenium chloride (II), HEK293, human embryonic kidney 293 cells, KGDHC, α-ketoglutarate dehydrogenase complex, MSE, mannitol/sucrose/EGTA, RET, reverse electron transfer, ROS, reactive oxygen species, SMP, submitochondrial particle

## Abstract

Impairments in mitochondrial energy metabolism have been implicated in human genetic diseases associated with mitochondrial and nuclear DNA mutations, neurodegenerative and cardiovascular disorders, diabetes, and aging. Alteration in mitochondrial complex I structure and activity has been shown to play a key role in Parkinson's disease and ischemia/reperfusion tissue injury, but significant difficulty remains in assessing the content of this enzyme complex in a given sample. The present study introduces a new method utilizing native polyacrylamide gel electrophoresis in combination with flavin fluorescence scanning to measure the absolute content of complex I, as well as α-ketoglutarate dehydrogenase complex, in any preparation. We show that complex I content is 19 ± 1 pmol/mg of protein in the brain mitochondria, whereas varies up to 10-fold in different mouse tissues. Together with the measurements of NADH-dependent specific activity, our method also allows accurate determination of complex I catalytic turnover, which was calculated as 10^4^ min^−1^ for NADH:ubiquinone reductase in mouse brain mitochondrial preparations. α-ketoglutarate dehydrogenase complex content was determined to be 65 ± 5 and 123 ± 9 pmol/mg protein for mouse brain and bovine heart mitochondria, respectively. Our approach can also be extended to cultured cells, and we demonstrated that about 90 × 10^3^ complex I molecules are present in a single human embryonic kidney 293 cell. The ability to determine complex I content should provide a valuable tool to investigate the enzyme status in samples after *in vivo* treatment in mutant organisms, cells in culture, or human biopsies.

Mitochondrial complex I (Enzyme Commission number: 1.6.5.3) is a key component of the respiratory chain that catalyzes oxidation of matrix NADH by ubiquinone, which is coupled with proton translocation across the inner mitochondrial membrane (see Refs. (1, 2) for reviews). The enzyme contains one molecule of FMN tightly bound to the 51 kDa (NDUFV1) subunit that was first determined biochemically (3, 4, 5, 6) and then confirmed by structural data (7, 8, 9). The FMN molecule serves as the primary electron acceptor for NADH and transfers electrons downstream to the chain of FeS clusters. FMN-deficient complex I cannot catalyze reactions of the direct (10, 11) or reverse (12) physiological reaction and is also not active in reactive oxygen species (ROS) generation (12, 13). Impairments of complex I function at that first redox step are associated with several pathophysiological states and genetic diseases (14). Several human pathological mutations are localized in the NDUFV1 subunit close to the nucleotide-binding site and most likely can affect FMN redox properties, binding affinity, or stability of the enzyme (15, 16, 17, 18). Loss of mitochondrial complex I integrity and activity was found in tissues of patients with Parkinson's disease (19, 20), which plays a role in epileptogenesis (21). Recently, we found that mitochondrial impairment during brain ischemia/reperfusion is due to inactivation of complex I *via* dissociation of FMN from the holoenzyme (13, 22) (see Ref. (23) for review). Loss of flavin from the enzyme was also observed during reperfusion of ischemic liver and kidneys used for transplantation (24, 25). Rapidly rising interest in biomedically oriented studies of mitochondrial complex I demands a reliable procedure for fast and accurate determination of enzyme content in a given sample.

The method presented here allows quantitative analysis of complex I–bound FMN content and is based on native polyacrylamide gel electrophoresis and fluorimetric scanning. This approach has great potential use for the determination of absolute content and catalytic turnover number of complex I in preparations obtained from genetic mutants, tissues *ex vivo*, and human biopsies. Our study provides a valuable tool for utilizing complex I content in homogenates and cells as a valid biomarker.

## Results

### High-resolution clear native electrophoresis of intact brain mitochondria and flavin fluorescence

**Figure 1:**
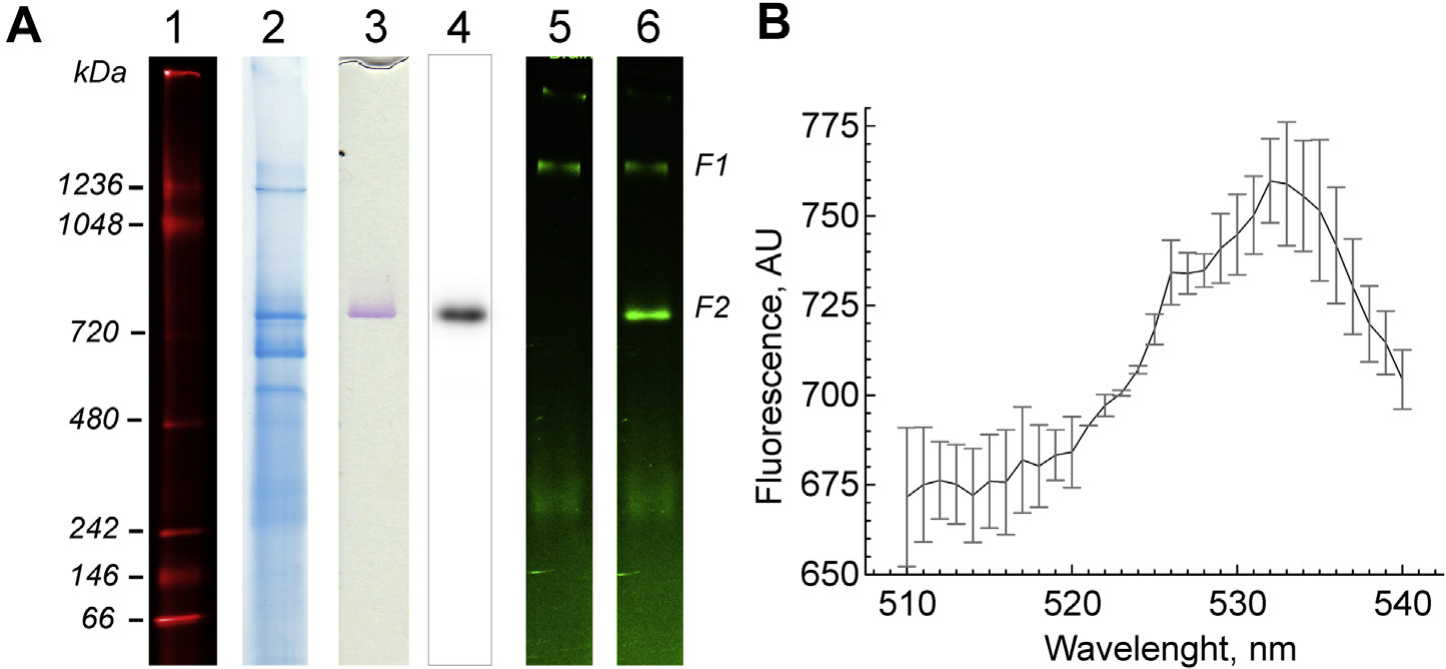
**Specific features of an hrCN gel using DDM-solubilized intact brain mitochondria.***A*, mouse brain mitochondria were solubilized using a DDM/protein ratio of 3.6 g/g. Identical aliquots (15 μg) were loaded on a polyacrylamide gradient gel (3–12%). hrCNE was performed as described (76). *Lane 2*, Coomassie-stained gel strips showing the presence of complexes (I–V). *Lane 1*, the position of fluorescently labeled molecular weight markers in kilodalton is shown to the *left*; *lane 3*, complex I in-gel activity strip; *lane 4*, complex I immunodetection by Western blot with antibody against NDUFS8 subunit; *lanes 5* and *6*, flavin fluorescence signal from the same strip before (5) and after (6) 20 min incubation with 20% SDS, respectively. Mass spectrometric protein identifications for flavin-containing bands F1 and F2 from an untreated hrCN gel are provided in Table 1. *B*, averaged fluorescence emission spectrum (excitation at 450 nm) of the extract of flavin-containing band F2 (mean ± SEM, n = 3). DDM, *n*-dodecyl-β-d-maltoside.

A typical Coomassie-stained high-resolution clear native electrophoresis gel is shown in Figure 1*A*, *lane 2*. The complex I band was stained by an “in-gel” NADH:nitro tetrazolium reaction catalyzed by the enzyme (Fig. 1*A*, *lane 3*). Flavin fluorescent scanning of a gel (excitation of 473 nm/emission of 530 nm) showed bright flavin fluorescence in the upper part of the gel in a band of apparent molecular weight 2.5 to 2.7 MDa (F1 band) (Fig. 1*A*, *lane 5*). Flavin of complex I was not detected in the native gel because enzyme-bound FMN fluorescence is quenched in the intact enzyme (26, 27, 28). However, complex I FMN fluorescence can be revealed after denaturation of the complex I molecule. After the addition of 20% SDS directly on top of the gel, a strong flavin fluorescence signal appeared as a single band corresponding to the position of complex I (∼850 kDa, F2 band). The MS/MS identification of FMN-containing bands F1 and F2 is given in Table 1. MS/MS analysis indicates that band F1 corresponds to the flavin adenine dinucleotide (FAD)–containing dihydrolipoyl dehydrogenase (DLD) subunit of α-ketoglutarate dehydrogenase complex (KGDHC) and band F2 to mitochondrial complex I, which FMN fluorescence revealed only after denaturation of the enzyme. The characteristic fluorescence emission spectrum of flavin was found in extracts from the F2 band (Fig. 1*B*).

**Table 1:** List of most abundant mitochondrial proteins identified by LC–MS/MS in the flavin fluorescent gel bands F1 and F2 resolved by hrCNE from Figure 1

Number	Protein name	Molecular weight (kDa)	Access number (National Centerfor Biotechnology Information)	Protein spectrum count^a^	
				F1	F2
1	2-oxoglutarate dehydrogenase	118.18	Q60597-3	415	6
2	Dihydrolipoyllysine-succinyltransferase	48.99	Q9D2G2	277	1
3	OGDLH protein	114.54	B2RXT3_MOUSE	234	3
4	MICOS complex subunit Mic60	82.93	Q8CAQ8-2	234	70
5	ADP/ATP translocase 2	32.93	ADT2_MOUSE	90	32
6	Dihydrolipoyl dehydrogenase	54.27	DLDH_MOUSE	53	5
5	NADH-ubiquinone oxidoreductase 75 kDa	79.70	Ndufs1	33	328
6	ATP synthase subunit beta	59.75	Atp5f1b	79	242
7	NADH dehydrogenase flavoprotein 1	49.91	Ndufv1	18	112
8	NADH dehydrogenase FeS protein 2	52.63	Ndufs2	20	107
9	NADH dehydrogenase subunit 10	40.60	Ndufa10	15	93
10	NADH dehydrogenase subunit 9	42.12	Ndufa9	16	71

a Protein spectrum count was normalized by the total spectral count. The detailed MS data are provided in Table S1.

**Figure 2:**
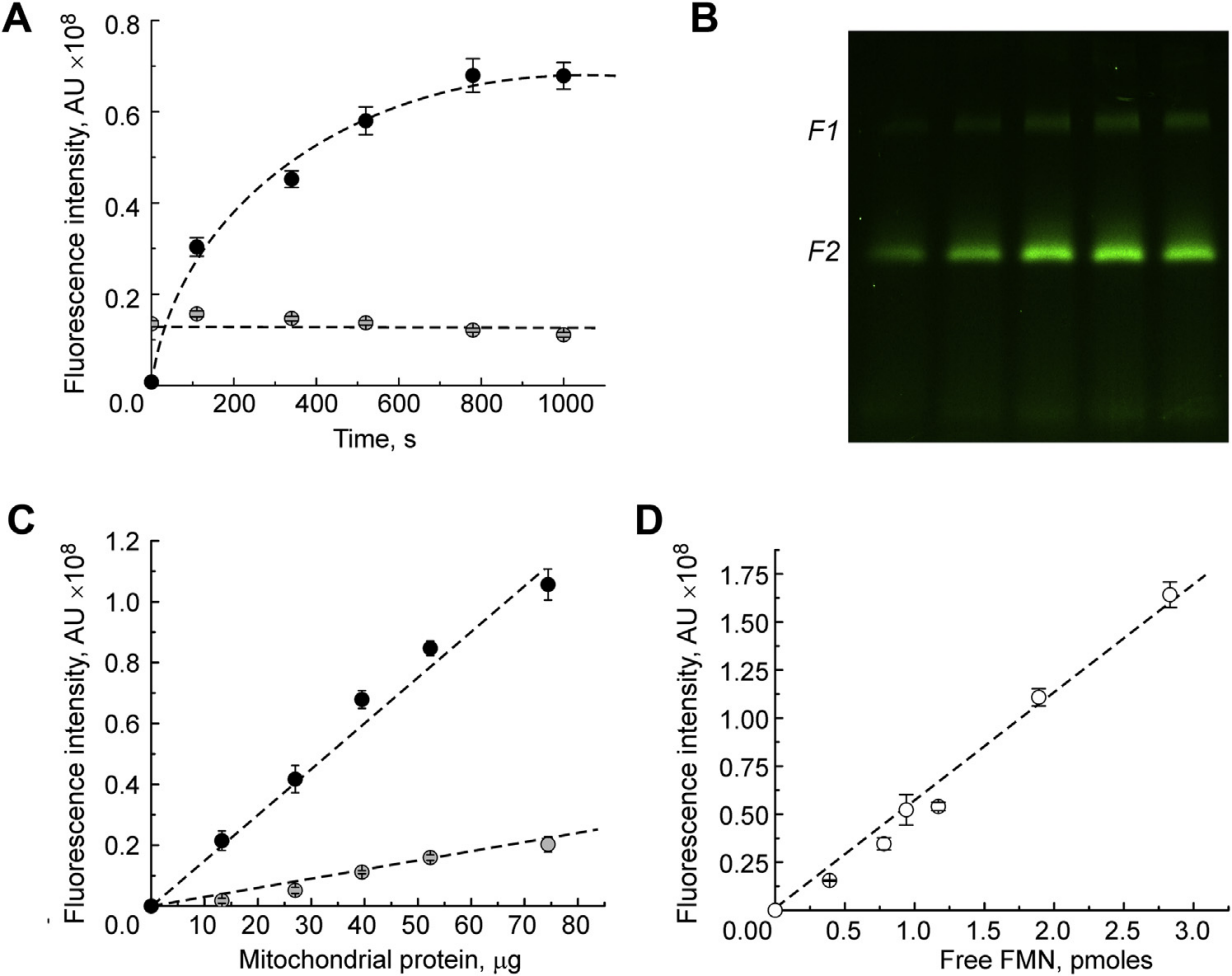
**Properties of flavin fluorescence signal from an hrCN gel of DDM-solubilized brain mitochondria.***A*, time dependence of the upper KGDHC and lower complex I (*gray and black circles*, respectively) flavin-containing bands (as in Fig. 1) after 20% SDS was applied on the gel surface and repetitive fluorescent scans performed, protein load 40 μg. *B*, representative fluorescence image of an SDS-treated hrCN gel showing flavin fluorescence intensity at different protein loads (13–74 μg per well from *left* to *right*). *C*, dependence of the fluorescence intensity of the KGDHC and complex I bands upon protein load on the gel. Values are mean ± SEM (n = 4). *D*, calibration curve for quantitative analysis made of standard FMN solution applied directly on the gel surface and intensity of fluorescent spots measured (mean ± SEM, n = 3). DDM, *n*-dodecyl-β-d-maltoside; KGDHC, α-ketoglutarate dehydrogenase complex.

Denaturation-induced FMN fluorescence is not instantaneous and takes at least 15 min for the signal to fully develop for 1-mm gels (Fig. 2*A*). Longer time exposure resulted in broadening of the band, most likely because of the diffusion of the cofactor through the porous gel. The intensity of the upper band F1 does not change upon SDS treatment. The fluorescent scan of an SDS-treated gel with different amounts of loaded protein is shown in Figure 2*B* (range of 13–74 μg protein/well). Densitometric analysis of the bands' intensity showed a linear dependence of the band fluorescence on the amount of loaded protein (Fig. 2*C*).

### Quantitative determination of complex I content in preparation of brain mitochondria

The flavin fluorescence signal from complex I (FMN) or KGDHC bands in gels can be quantitatively estimated by calibration with a known amount of flavin added directly on the gel surface during scanning. The sensitivity of the Typhoon instrument was estimated to be 20 to 40 fmol of FMN, and the fluorescence intensity depended linearly on the amount of FMN added in the range of 0.3 to 3 pmol (Fig. 2*D*). Based on the calibration, we calculated absolute content of complex I and KGDHC in our brain mitochondria samples. Complex I contains one FMN per molecule of enzyme, therefore the calculated complex I content in intact brain mitochondria was determined as 18.6 ± 0.9 pmol/mg protein (n = 4). FAD fluorescence is only 15% of that of FMN (29, 30), and from our calibration, the calculated content of FAD of DLD (upper F1 band in Fig. 1*A*) was determined as 65.0 ± 4.7 pmol FAD/mg protein (Table 2).

**Table 2:** Analysis of complex I content and catalytic turnover number in whole tissue homogenates from different mouse tissues, intact mouse brain mitochondria, bovine mitochondria, and SMP

Preparation type	KGDHC FAD content pmol/mg protein	Complex I FMN content pmol/mg protein	NADH:HAR^a^		NADH:Q_1_^a^	
			Activity μmol × min^−1^ × mg^−1^	Turnover^b^ ×10^4^, min^−1^	Activity μmol × min^−1^ × mg^−1^	Turnover^b^ ×10^4^, min^−1^
Brain	17 ± 1	10 ± 1	0.91 ± 0.06	8.96 ± 1.00	0.105 ± 0.004	1.03 ± 0.10
Heart	38 ± 8	50 ± 5	5.39 ± 0.40	10.9 ± 1.29	0.229 ± 0.022	0.46 ± 0.06
Liver	ND	6 ± 1	0.75 ± 0.03	12.1 ± 1.15	0.037 ± 0.002	0.60 ± 0.06
Kidneys	16 ± 8	26 ± 3	1.62 ± 0.03	6.28 ± 0.80	0.125 ± 0.005	0.48 ± 0.06
Muscle	50 ± 3	20 ± 1	1.60 ± 0.10	7.87 ± 0.71	0.076 ± 0.010	0.37 ± 0.05
MB Mt^c^	65 ± 5	19 ± 1	1.62 ± 0.04	8.68 ± 0.47	0.21 ± 0.02	1.06 ± 0.03
BH Mt^c^	123 ± 9	51 ± 4	4.46 ± 0.16	8.80 ± 0.74	0.135 ± 0.007	0.27 ± 0.02
BH SMP^c^	ND	70 ± 6	5.86 ± 0.12	8.43 ± 0.80	0.071 ± 0.005	0.10 ± 0.01

Abbreviation: ND, not detected.

a Activities were assayed accordingly to the Experimental procedures section at 25 °C in buffer made of 125 mM KCl, 0.02 mM EGTA, 20 mM Hepes–KOH (pH 7.5) and either 1 mM HAR or 40 to 50 μM Q_1_. About 100 μM of NADH was added to 2 to 30 or 50 to 400 μg/ml protein for HAR and Q_1_ reactions, respectively. Values are given in μmol NADH × min^−1^ × mg protein^−1^.

b Enzyme catalytic turnover (*k*_cat_) was calculated as moles of NADH oxidized per minute per mole of enzyme by dividing activity values in μmol × min^−1^ × mg protein^−1^ by enzyme content in micromoles of complex I per milligram of protein.

c Intact mitochondria from mouse brain (MB Mt), bovine heart (BH Mt), and bovine heart SMP (BH SMP).

Based on complex I content values, it is possible to determine the enzyme turnover number in NADH-dependent activities of complex I in brain mitochondria. After solubilization of mitochondrial membranes with *n*-dodecyl-β-d-maltoside (DDM) and separation of supernatant, NADH:hexaammineruthenium chloride (II) (HAR) and NADH:Q_1_ oxidoreductase activities were measured before applying onto an hrCN gel (Table 2).

### Direct detection of FMN loss from mitochondrial complex I

**Figure 3:**
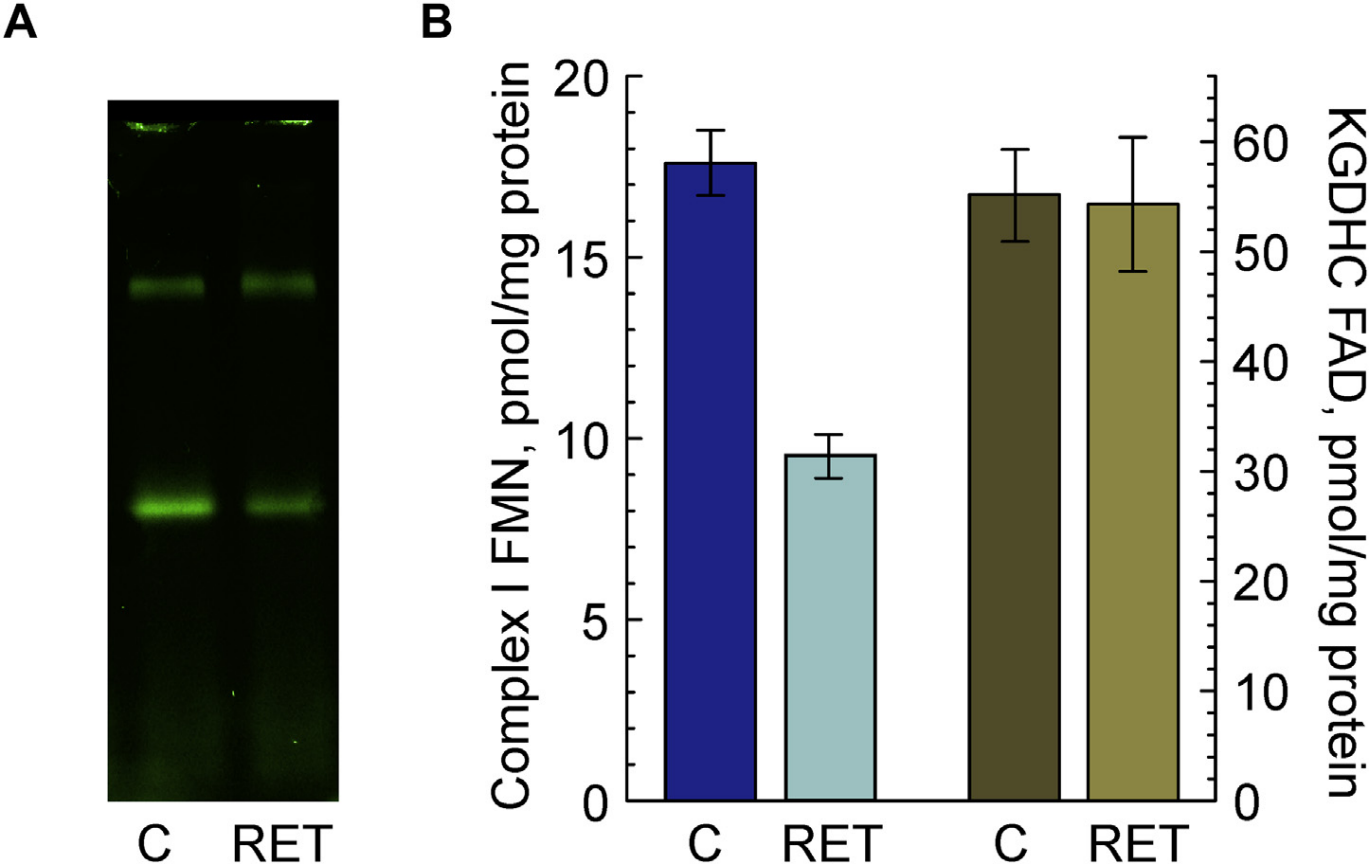
**Effect of reverse electron transfer (RET) in preparation of intact brain mitochondria on the content of complex I FMN and FAD of KGDHC.***A*, representative flavin fluorescence image of an hrCN gel showing signal from DDM-solubilized brain mitochondria before (C) and after 20 min of succinate oxidation (RET). Brain mitochondria (0.3–0.4 mg/ml of protein) were incubated in the presence of 5 mM succinate and 1 mM glutamate in nonphosphorylating conditions as described in the Experimental procedures section. After incubation, mitochondria were washed by centrifugation, solubilized by DDM as in Figure 1, and mitochondrial complexes were separated by hrCNE. *B*, quantitative estimation of complex I FMN (*blue bars*, *left vertical axis*) and KGDHC FAD (*olive bars*, *right vertical axis*). *Light bars* show flavin content after 20 min of incubation in RET-like conditions (mean ± SEM, n = 3). DDM, *n*-dodecyl-β-d-maltoside; FAD, flavin adenine dinucleotide; KGDHC, α-ketoglutarate dehydrogenase complex.

In the past, we showed that brain mitochondrial complex I can lose its natural cofactor FMN when incubated in conditions of so-called reverse electron transfer (RET) (12, 13, 22). In that state, electrons from succinate are directed to ubiquinone, reducing it to ubiquinol, and electrons are then transferred to complexes III and IV and oxygen is reduced to water. This generates a proton-motive force across the inner mitochondrial membrane, and a small fraction of electrons from ubiquinol is pushed upstream reducing all redox centers of complex I. Maintaining complex I FMN in the reduced state results in dissociation of FMNH_2_ from its binding site (12, 13, 22). To validate our method, we tested if this treatment results in a decreased complex I-associated FMN fluorescence signal. A typical flavin fluorescent gel of solubilized brain mitochondria before and after incubation in RET conditions is shown in Figure 3*A*. About 20 min of incubation in RET-like conditions resulted in a dramatic decrease of the complex I-associated FMN signal but had no effect on FAD from KGDHC.

### Quantitative determination of complex I content and enzymatic turnover number in whole tissue homogenates

**Figure 4:**
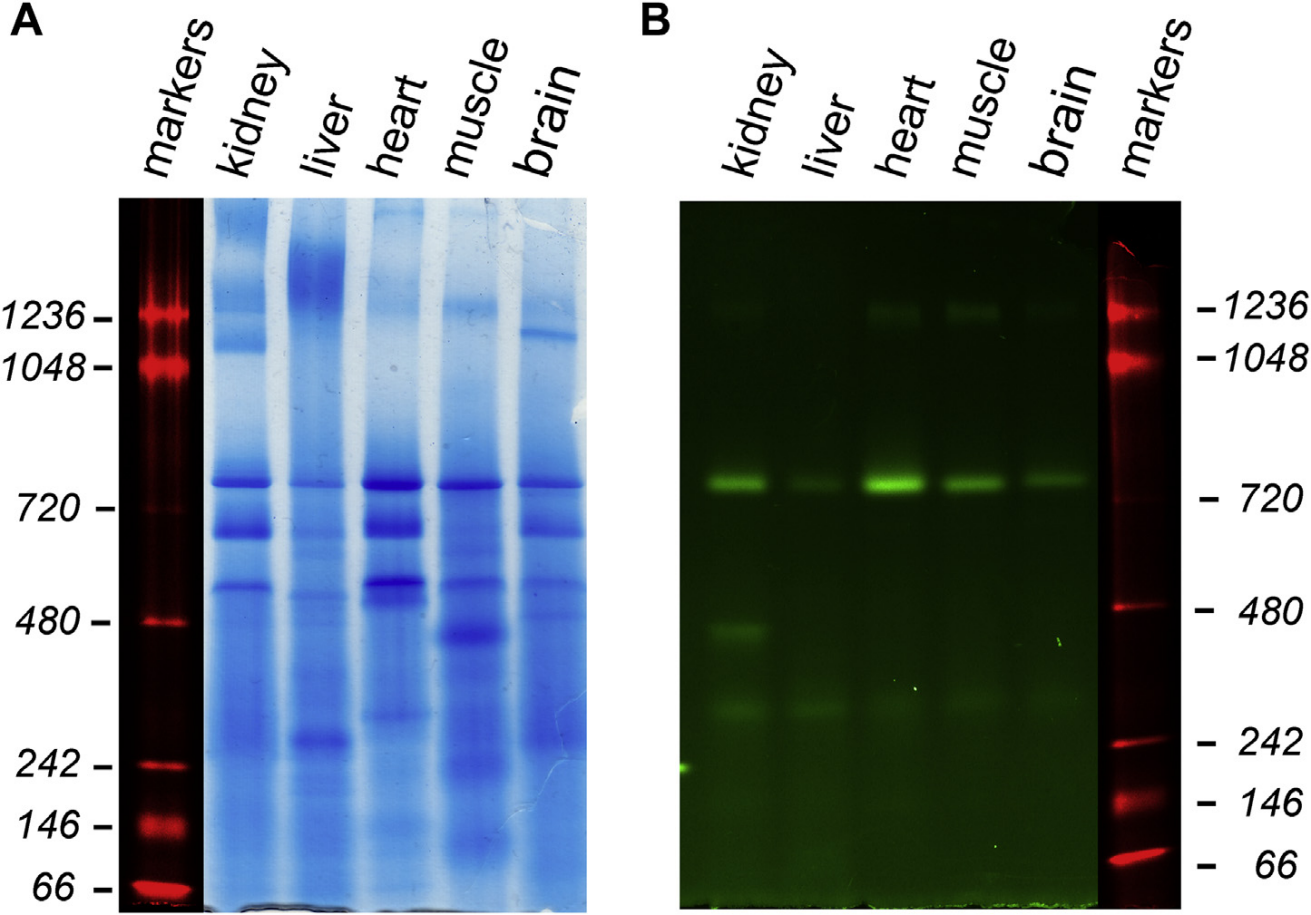
**Complex I flavin detection in DDM-solubilized whole tissue homogenates from different mouse organs.** Homogenates were prepared, treated with DDM as described in the Experimental procedures section, and identical aliquots (30 μg of protein) were loaded on an hrCN gel. Two gels were run in the same conditions and then treated separately for Coomassie protein stain (*A*) and fluorescent detection of flavin (*B*). Fluorescently labeled markers are shown in both gels from the *left* and *right* (*red*). DDM, *n*-dodecyl-β-d-maltoside.

The quantification of various mitochondrial proteins gives insight into the function and role of mitochondria in different tissues. It was of interest to find out whether this approach can be used for the measurements of complex I content and catalytic turnover number in different tissues without isolation of mitochondria first. We developed a fast and an accurate method based on whole tissue homogenate analysis. Flavin fluorescence of a representative gel containing protein solubilized from the brain, heart, liver, muscle, and kidneys homogenate is shown in Figure 4.

Knowing the turnover number (*k*_cat_, catalytic rate constant) of an enzyme is essential for understanding its regulation and function. By determining complex I content and specific activity, we calculated complex I catalytic turnover number in NADH:HAR and NADH:Q_1_ reductase reactions of complex I in homogenates from different tissues (Table 2). As expected, the highest complex I content was found in heart tissue, and values were strikingly similar between mouse heart and isolated bovine heart mitochondria. Moreover, while complex I content was different for mouse brain homogenate and isolated brain mitochondria, the turnover values were the same.

### Estimation of complex I content in preparations from different sources

Bovine heart mitochondria and submitochondrial particles (SMPs) are the classical object of study in bioenergetics; therefore, we determined their complex I content and enzymatic turnover number (Table 2). Among all studied preparations, SMPs have the highest complex I content (70 ± 6 pmol/mg protein).

**Figure 5:**
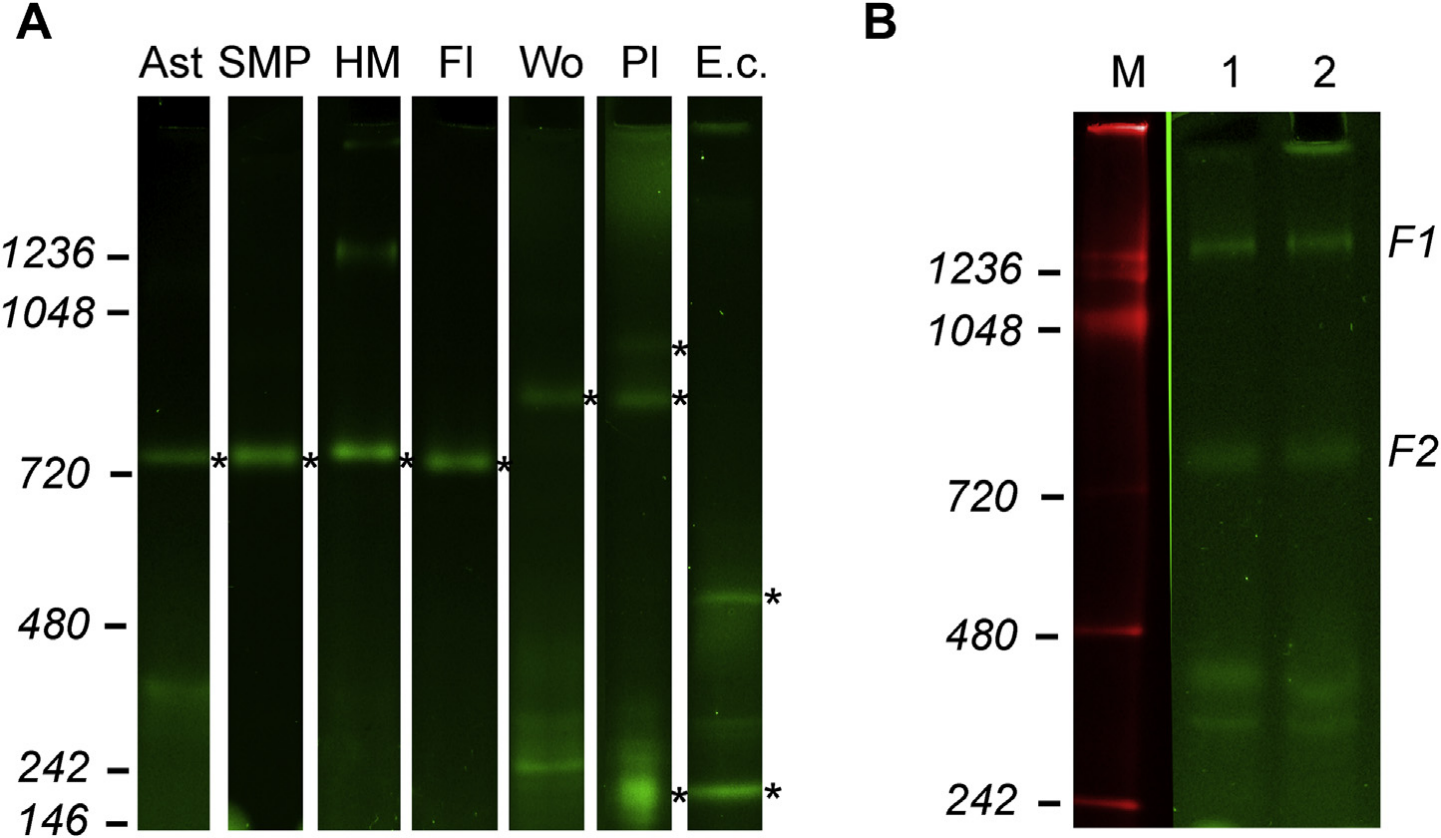
**Complex I FMN detection from different sources.***A*, membrane preparations obtained as described in the Experimental procedures section and solubilized using 3.6 g DDM/g protein for mitochondria from primary mouse astrocytes (25 μg of protein, Ast), submitochondrial particles (10 μg of protein, SMP), and mitochondria from bovine heart (10 μg of protein, HM), *Drosophila melanogaster* (10 μg of protein, Fl), *Lumbricus terrestris* (25 μg of protein, Wo), and *Escherichia coli* subacterial vesicles (30 μg of protein, E.c.). Plant mitochondria from *Vigna radiata* were solubilized with 2 g of digitonin/g protein, and 45 μg was applied (lane Pl). hrCNE and complex I FMN determination were performed as in Figure 2. Flavin fluorescent bands appeared after SDS treatment are labeled with an *asterisk* (∗). *B*, total homogenate prepared with cultured HEK293 cells was solubilized with DDM (3.6 g/g of protein, 3.2 × 10^5^ cells/well) and loaded on hrCN gel after (1) or without (2) centrifugation as explained in the Results section. KGDHC and complex I bands are marked F1 and F2, respectively. DDM, *n*-dodecyl-β-d-maltoside; HEK293, human embryonic kidney 293 cells; KGDHC, α-ketoglutarate dehydrogenase complex.

Finally, we validated our method using membrane preparations from different organisms and primary and cultured cells. As shown in Figure 5, SDS treatment of an hrCN gel resulted in the appearance of a single complex I FMN band (apparent molecular weight of 850–1000 kDa) in preparations of bovine, *Drosophila*, earthworm, and plant mitochondria, as well as primary astrocytes and cultured human embryonic kidney 293 (HEK293) cells. Note that complex I in plants and worms is slightly larger than the mammalian enzyme probably because of the presence of extra subunits in plant complex I (31, 32) or tight association of the enzyme with complex III at a low DDM/protein ratio as was shown for the *Caenorhabditis elegans* enzyme (33). Bacterial complex I is more fragile than the mitochondrial enzyme (34), and in our hands, separation of *Escherichia coli* membranes resulted in two complex I flavin bands of around 550 and 170 kDa (also displaying in-gel activity, not shown). The low molecular weight band corresponds to the NADH dehydrogenase module of *E. coli* complex I (subunits NuoE, F, and G; total molecular weight of 170 kDa) and was observed previously after detergent treatment complex I preparations at pH >6 (35) or as an assembly intermediate (36).

Cultured cells are an established pipeline for studying the assembly of complex I and clinically relevant mutations (37, 38, 39, 40); therefore, we tested the versatility of our approach using the HEK293 cell line (Fig. 5*B*). Harvested cells were homogenized, solubilized using DDM, and the resulting lysate was either applied directly on an hrCN gel or centrifuged, and only the supernatant was loaded in accordance to the original protocol. For both approaches, we obtained very close values of FMN or FAD content. For the DDM-solubilized total cell suspension (no centrifugation), the values of 0.152 ± 0.003 pmol FMN/10^6^ cells and 1.00 ± 0.08 pmol FAD/10^6^ cells were determined for complex I and KGDHC, respectively.

## Discussion

Two main strategies are most commonly used when the absolute content of a membrane enzyme such as complex I is to be measured. One is to carefully titrate the activity of the enzyme with a specific tightly bound inhibitor, where complex I content is determined as the intersection points from the linear graphs of the residual activity *versus* the amount of inhibitor added (per milligram of protein). The main assumption for this method is 1:1 stoichiometry of inhibitor binding. Another approach is to treat a membrane preparation with a denaturing agent, and after protein precipitation, fluorometrically determine soluble noncovalently bound FMN assuming that it originates from complex I (5, 13, 22). However, if a membrane preparation from an unfamiliar source is used, there is a considerable risk of overestimation of complex I content because of the presence of other proteins contributing to liberated noncovalently linked flavin, especially in prokaryotic membranes (41). A relative estimation *via* immunochemical labeling by the Western blot approach requires rigorous optimization and does not produce reliable quantitative values. Therefore, the aforementioned strategies suffer from a number of disadvantages. The method presented here is based on determination of FMN that belongs only to complex I and for the first time allows accurate determination of absolute content of the enzyme.

Mitochondria contain a great number of flavoenzymes bearing either FMN or FAD as cofactors. The mammalian flavoproteome contains 15 FMN-containing proteins with only two enzymes localized in mitochondria and carrying a noncovalently bound FMN (42). Since the pioneering work of Britton Chance (43, 44, 45), flavin fluorescence has been an extremely useful tool in the study of mitochondrial energy metabolism. Flavin fluorescence provides a rapid and an accurate measurement of the redox state of mitochondrial enzymes that is suitable for biochemical or imaging analysis and can be utilized in clinical settings (24, 25, 46, 47, 48). In intact mitochondria, most of the protein-bound flavin is considered to be nonfluorescent except FAD of DLD of α-ketoglutarate and pyruvate dehydrogenase complexes and electron-transferring flavoprotein of oxidation of fatty acids (26, 49, 50, 51). At the same time, complex I-bound FMN is practically nonfluorescent because of quenching of the flavin upon binding to the apoenzyme.

Our method of complex I FMN detection utilizes the measurements of flavin fluorescence intensity after denaturation of the enzyme separated by native gel electrophoresis and provides accurate values of absolute complex I content. SDS was used as a denaturing agent and applied directly on the surface of a gel to induce complex I denaturation and liberation of FMN. Time is required for the complex I-specific FMN fluorescence signal to develop (Fig. 2*A*), indicating gradual denaturation of the enzyme inside the gel. With time, however, the released FMN diffuses within the gel pores, and fluorescence intensity as well as band sharpness decrease. In our experience, 15 to 20 min of exposure to 20% SDS is enough for the detection of total FMN in the band.

The value of 18.6 ± 0.9 pmol FMN/mg of protein in intact brain mitochondria found in this study is almost equivalent to the values determined for the same preparation using acid extraction in our previous study (17–21 pmol FMN/mg of protein, (13)). The obtained value of complex I content in bovine SMP (70 ± 6 pmol FMN/mg protein) is very close to the complex I content derived from ^14^C-labeled piericidin A titer determined in the pioneering work of the Thomas Singer group (around 70 pmol/mg of protein) (52) but lower than the rotenone titer (110–200 pmol/mg protein) (53, 54). The latter can be explained by the fact that two inhibitor molecules bind to complex I in the recently determined structure of the mammalian enzyme (55).

Our method also enables detection of FAD originating from the DLD subunit of KGDHC of the Krebs cycle. Protein denaturation is not required to reveal flavin fluorescence, and SDS treatment does not change the intensity of the FAD signal of the KGDHC band. This is in good agreement with the observation that, unlike FMN of complex I, FAD fluorescence is not quenched upon binding to the polypeptide (56). In the preparation of mouse brain mitochondria, FAD content was estimated as 65.0 ± 4.7 pmol FAD per milligram of protein. It has been shown recently that a fully assembled KGDHC contains 12 DLD subunits per assembled complex (57). Therefore, the calculated stoichiometry of KGDHC is equivalent to 5.4 ± 0.4 pmol KGDHC/mg of protein. This indicates that in mouse brain mitochondria, the complex I:KGDHC ratio is close to 3:1. This ratio varies greatly in homogenates obtained from different organs probably indicating a difference in mitochondrial population density and tissue metabolic profile. However, quantitative estimation of KGDHC content based on FAD fluorescence observed by our method should be treated with caution. In frozen/thawed solubilized membranes, the intensity of the band may vary considerably pointing at the disintegration of KGDHC upon sample processing.

Knowledge of the absolute content of complex I is a prerequisite for the determination of the turnover number (*k*_cat_), which is an important parameter to evaluate an enzyme's efficiency and stability. The value determined for the mouse brain enzyme in NADH:Q_1_ reductase reaction (Table 2; 10^4^ min^−1^ = 176 s^−1^) is very similar to the values for purified *Paracoccus denitrificans* NADH dehydrogenase (140 s^−1^ (58), 186 s^−1^ (41)), reconstituted enzyme from *Yarrowia lipolytica* (110–130 s^−1^, (59)), and membrane-bound bovine enzyme (110–150 s^−1^ (60)). As expected, the turnover number for NADH oxidation by artificial acceptor HAR, which reacts at the N module of complex I, is significantly higher than the physiological NADH:Q_1_ reaction (41, 61, 62).

We further validated our approach by comparison of complex I FMN content in brain mitochondria incubated in conditions of RET, when a small fraction of electrons from succinate is driven by the membrane potential upstream to complex I FMN (63). RET supports the highest rate of ROS generation in intact mitochondria, and complex I-reduced flavin has been shown as the main site of ROS production (12, 64, 65, 66, 67, 68, 69, 70, 71, 72). From the thermodynamic analysis of bovine complex I (73), it follows that oxidized FMN would have more than four orders of magnitude higher affinity for its specific binding site than reduced flavin. We have previously reported that incubation in RET conditions results in FMN dissociation from the native enzyme in brain mitochondria (12, 13). Indeed, 20-min incubation of intact mitochondria in RET conditions led to a twofold decrease of FMN in the complex I band without apparent effect on the FAD signal from KGDHC (Fig. 3).

This native gel electrophoresis–fluorimetric scan approach has great potential use for a quantitative determination of absolute content and catalytic turnover number of complex I in preparations obtained from genetic mutants, tissues *ex vivo*, and human biopsies. Therefore, we developed a procedure for the determination of complex I absolute content that does not require mitochondrial isolation. For different mouse tissues, we optimized conditions for homogenates preparation and solubilization and were able to reliably quantify FMN content and turnover number for complex I in NADH:HAR and NADH:Q_1_ reductase reactions. Interestingly, the values vary significantly probably indicating either tissue-specific difference of complex I (74, 75) or a disparate effect of DDM solubilization on homogenates from different organs.

Our results obtained with cultured HEK293 cells require special consideration. The sample preparation procedure requires centrifugation of solubilized membranes after the addition of detergent (76, 77). We should stress that we applied DDM-solubilized whole cell lysate on the hrCN gel, without centrifugation after detergent treatment. Surprisingly, it did not significantly affect the electrophoretic separation process or band intensity, indicating that most of the mitochondrial complex I was solubilized and entered the gel. From the obtained value of complex I content of 1.5 pmol/10^7^ cells, we calculated that a single HEK293 cell contains approximately 90 × 10^3^ molecules of mitochondrial complex I.

## Conclusions

The method reported here can be recommended as a valuable tool for accurate estimation of complex I content in samples from various sources. We have provided some examples of the versatility of this technique, which can be used for the analysis of proteins with noncovalently bound flavin cofactors to identify assembly intermediates (including soluble), specific ligands, degradation dynamics, and other parameters. This quantitative information will be extremely useful for any biochemical characterization of a given complex I preparation, including ones for crystallization and cryomicroscopy. This is a fast, cheap, and reliable method that will be of great help for the many groups that study complex I using cultured cells. Potentially, complex I content assay can be used as a valid biomarker for mitochondria-related pathologies.

## Experimental procedures

### Sources of chemicals

Most of the chemicals were purchased from Sigma, including mannitol (catalog no. 63559), sucrose (catalog no. 84097), essentially fatty acid–free bovine serum albumin (BSA; catalog no. A6003), NADH (catalog no. N8129), hexaammineruthenium (III) chloride (catalog no. 262005), Q_1_ (catalog no. C7956), DDM (catalog no. S#D4641), and triphenyl-tetrazolium chloride (catalog no. T8877). Pierce bicinchoninic acid protein assay kit (catalog no. 23225), Amplex UltraRed (catalog no. A36006), horseradish peroxidase (catalog no. 012001), 3 to 12% acrylamide gradient gel, 8 × 8 cm (catalog no. BN1001BOX), NativeMark unstained protein standards (catalog no. LC0725), and PageBlue Protein Staining Solution (catalog no. 24620) were from Thermo Fisher Scientific. Alamethicin (catalog no. 11425) and atpenin A5 (catalog no. 11898) were from Cayman Chemical.

### Isolation of mitochondrial and bacterial membranes

Intact brain mitochondria were isolated from C57BL6/J mice by differential centrifugation with digitonin treatment (12). Intact heart mitochondria were isolated (78). Bovine heart mitochondria and SMPs were prepared according to the standard procedure (79). Mitochondria from earthworms (*Lumbricus terrestris*) and sprouting mung beans (*Vigna radiata*) were isolated essentially as described (80, 81), respectively, except mannitol/sucrose/EGTA (MSE) isolation medium (225 mM mannitol, 75 mM sucrose, 20 mM Hepes–KOH, 1 mM EGTA, 1 mg/ml BSA, and pH 7.4) was used. Isolation of mitochondria from *Drosophila melanogaster* wing muscles was performed (82). *E. coli* subbacterial membranes were isolated essentially (83), except 0.5 M aminocaproic acid was used in the isolation buffer.

### Preparation of HEK293 cultured cells and primary astrocytes

HEK293 cells were grown and harvested as described (84). Mouse brain cortex primary astrocytes were isolated from the mixed glial culture (85).

### Preparation of homogenates

Brain, muscle, heart, kidney, and liver tissues were extracted from mice and suspended in MSE with tissue:buffer ratio 1:10 (w/w). Samples were homogenized with a Potter homogenizer for 1 to 3 min at 1450 rpm. Tissue homogenates were centrifuged at a low-speed (500*g*, 4 min), and the resulting supernatants were pelleted at 10,000*g* for 10 min to obtain crude mitochondria.

Primary or cultured cells were washed with PBS (500*g*, 4 min) and suspended in a hypotonic isolation buffer (threefold diluted MSE, supplemented with 1.7 mM aminocaproic acid) with tissue:buffer ratio 1:10 (w/w). Homogenization was performed using 40 up and down strokes with a Potter homogenizer at 1450 rpm. Cell permeability was tested with trypan blue, and another round of homogenization was repeated if necessary to ensure permeabilization. After 500*g* (3 min) centrifugation, the supernatant was aliquoted and pelleted at 10,000*g* for 10 min. All operations were conducted at 4 °C.

### Intact mitochondria treatment in RET-like conditions

Mitochondria (0.9–1.2 mg of protein) were added to 2 ml of respiration buffer composed of 125 mM KCl, 0.02 mM EGTA, 20 mM Hepes–KOH, pH 7.5, 4 mM KH_2_PO_4_, 2 mM MgCl_2_, 2 mg/ml BSA, at 37 °C. To initiate RET-like conditions (12, 13), 5 mM succinate and 1 mM glutamate were added in nonphosphorylating conditions (state 2).

### Measurement of respiratory chain enzymes activities

All activities were measured spectrophotometrically using Molecular Devices SpectraMax M2 plate reader in 0.2 ml of assay buffer composed of 125 mM KCl, 20 mM Hepes–KOH (pH 7.5), 0.02 mM EGTA, at 25 °C.

NADH-dependent activities of complex I were assayed as oxidation of 100 μM NADH at 340 nm (ε_340nm_ = 6.22 mM^−1^ cm^−1^) in the assay buffer supplemented with either 1 mM HAR or 40 to 50 μM Q_1_. Mouse brain mitochondria activities were measured using 10 to 30 μg/ml for HAR and 100 to 125 μg/ml of DDM-solubilized brain mitochondria for Q reductase activities. For tissue homogenates, DDM-solubilized protein was added to concentration of 2 to 30 or 50 to 400 μg/ml for HAR and Q_1_ reductase activities, respectively.

### High-resolution clear native electrophoresis

Sample preparation for high-resolution clear native electrophoresis of mitochondrial membranes and tissues was essentially done as described (76). Briefly, mitochondria (400 μg) were resuspended in 50 μl solubilization buffer (50 mM imidazole, pH 7, 1 mM EDTA, and 0.5 M aminocaproic acid) and solubilized with DDM (3.6 g/g detergent to protein ratio). Tissue homogenates (1 mg) were resuspended in 35 μl solubilization buffer and solubilized with DDM (2 g/g). DDM-treated suspensions were centrifuged for 20 min at 20,000*g*, and the pellet was discarded. For HEK293 cells, DDM-solubilized protein was applied onto the gel without an additional centrifugation step. Supernatants were supplemented with 0.1% Ponceau S stain in 50% glycerol and loaded onto a 3 to 12% acrylamide gradient gel. NativeMark unstained protein standards were labeled with DyLight 680 *N*-hydroxysuccinimide ester and added to each gel. After native electrophoresis in a cold room, gels were analyzed either for complex I in-gel activity (76) or for flavin fluorescence in a Typhoon 9000 gel scanner (GE) using 473-nm laser and BPB1 filter (530-nm maximum, 30-nm bandpass) and for DyLight680 using 635 nm laser and LPR filter (665-nm long pass).

### Coomassie staining and in-gel activity

When necessary, gels were incubated with PageBlue Protein Staining Solution for 30 min and washed with Milli-Q water several times (or overnight) on a shaker.

For the in-gel activity assay of complex I, gels were placed in 10 ml of buffer containing 5 mM Tris–HCl, pH 7.4, 0.2 mM EDTA, and 3 mM nitro blue tetrazolium. About 150 μM NADH was added to the reaction, and after 3 to 5 min, gels were transferred to a fixing solution of 50% methanol and 10% acetic acid (76).

### Extraction of flavin from complex I band on hrCN gel

For extraction of flavin from an hrCN gel, 3 to 4 flavin-containing gel pieces (weight of around 15 mg) were excised, homogenized in 0.5 ml PBS, and incubated for 20 to 30 min under constant stirring. The gel suspension was separated by brief centrifugation for 5 min at 20,000*g* at 4 °C. The fluorescence emission spectrum was measured in the resulting supernatant using a 0.2-ml microcuvette (Starna Cells, Inc) and Hitachi F-7000 fluorospectrophotometer (excitation of 470 nm).

### Western blot analysis

Native gels were incubated for 20 min in a threefold gel volume of buffer (300 mM Tris, 100 mM acetic acid, 1% SDS, and pH 8.6). The gel was then placed between two plastic plates and stored for 40 min at 20 to 25 °C to spread SDS in the gel evenly and to denature proteins. Proteins were then electroblotted from the gel onto polyvinylidene difluoride membranes using SDS-free transfer buffer (150 mM Tris, 50 mM acetic acid, and pH 8.6). The proteins were transferred overnight (∼15 h), 4C, with the current set to 0.4 mA/cm^2^, and the voltage was limited to 15 V. The membrane was blocked with nonfat 5% milk in Tris-buffered saline with Tween-20. To detect complex I, we used an antibody against the S8 subunit of the mitochondrial membrane respiratory chain NADH dehydrogenase (complex I) (Rabbit monoclonal NDUFS8—C-terminal; Abcam; catalog no. ab180183: dilution 1: 2000; 2% milk; 2 h at room temperature). Horseradish peroxidase–conjugated secondary goat anti-rabbit Abs were used (Jackson Immunoresearch; catalog no. 111-035-144: dilution 1:10,000). Images of the blots were obtained using Fluorchem M Western Imaging System (Protein Simple) and processed with ImageJ software (NIH).

### MS/MS analysis

The band of interest was excised from an hrCN gel, and in-gel digestion was performed as previously described (86), with minor modifications. The gel slice was washed with 1:1 acetonitrile and 100 mM ammonium bicarbonate for 30 min and then dehydrated with 100% acetonitrile for 10 min until shrunk. Excess acetonitrile was then removed, and the slice was dried in speed vacuum at room temperature for 10 min. The gel slice was reduced with 5 mM DTT for 30 min at 56 °C in an air thermostat, cooled down to room temperature, and alkylated with 11 mM iodoacetamide for 30 min with no light. The gel slice was then washed with 100 mM of ammonium bicarbonate and 100% acetonitrile for 10 min each. Excess acetonitrile was removed and dried in a speed vacuum for 10 min at room temperature, and the gel slice was rehydrated in a solution of 25 ng/μl trypsin in 50 mM ammonium bicarbonate for 30 min on ice and digested overnight at 37 °C in an air thermostat. Digested peptides were collected and further extracted from the gel slice in extraction buffer (1:2 ratio by volume of 5% formic acid:acetonitrile) at high speed, shaking in an air thermostat. The supernatants from both extractions were combined and dried in a speed vacuum. Peptides were dissolved in 3% acetonitrile/0.1% formic acid.

Desalted peptides were injected in an EASY-Spray PepMap RSLC C18 50 cm × 75 cm ID column (Thermo Fisher Scientific) connected to an Orbitrap Fusion Tribrid (Thermo Fisher Scientific). Elution and separation of peptides were achieved at a nonlinear flow rate of 250 nl/min using a gradient of 5 to 30% of buffer B (0.1% [v/v] formic acid, 100% acetonitrile) for 110 min with the temperature of the column maintained at 50 °C during the entire experiment. The Thermo Scientific Orbitrap Fusion Tribrid mass spectrometer was used for peptide tandem mass spectroscopy (MS/MS). Survey scans of peptide precursors are performed from 400 to 1500 *m/z* at 120,000 full width at half maximum resolution (at 200 *m/z*) with a 2 × 10^5^ ion count target and a maximum injection time of 50 ms. The instrument was set to run in top speed mode with 3-s cycles for the survey and the MS/MS scans. After a survey scan, MS/MS was performed on the most abundant precursors, that is, those exhibiting a charge state from 2 to 6 of greater than 5 × 10^3^ intensity, by isolating them in the quadrupole at 1.6 Th. We used collision-induced dissociation with 35% collision energy and detected the resulting fragments with the rapid scan rate in the ion trap. The automatic gain control target for MS/MS was set to 1 × 10^4^, and the maximum injection time was limited to 35 ms. The dynamic exclusion was set to 45 s with a 10 ppm mass tolerance around the precursor and its isotopes. Monoisotopic precursor selection was enabled.

### LC–MS/MS data analysis

Raw mass spectrometric data were analyzed using the MaxQuant environment, version 1.6.1.0 (87) and Andromeda for database searches (88) at default settings with a few modifications (searched). The default is used for first search tolerance and main search tolerance (20 and 6 ppm, respectively). MaxQuant was set up to search with the UniProt *Mus musculus* ыtrain C57BL/6J proteome database (released March 7, 2021). MaxQuant performed the search trypsin digestion with up to two missed cleavages. Peptide, site, and protein false discovery rates were all set to 1% with a minimum of two peptides needed for identification. The following modifications were used for protein quantification: oxidation of methionine (M), acetylation of the protein N terminus, and deamination for asparagine or glutamine (NQ). Results obtained from MaxQuant were uploaded in Scaffold for data visualization.

### Statistical analysis

Gel fluorescence scans were analyzed using ImageLab software (Bio-Rad). Region of interest amplitude measurements were placed over the fluorescence band, and the intensity was calculated with background subtracted. Data analysis was performed using Microcalc Origin (version 8Pro). All data are mean ± SEM. Statistically significant differences are indicated (∗) when *p* < 0.05. Two-tailed *t* test or Mann–Whitney test was used to analyze intergroup differences between two groups. One-way ANOVA and Dunnet's test were used to compare groups after and before RET.

## Data availability

The MS raw files and result files used in this study have been deposited to the PRIDE repository (accession number: PXD028113). Other data supporting the findings of this article are available from the corresponding authors upon reasonable request.

## Dedication

Dedicated to the memory of our friend and colleague, Professor Andrei Vinogradov (Moscow State University, Russia), a devoted scholar of mitochondrial complex I who deceased on March 4, 2021.

## Supporting information

This article contains supporting information.

## Conflict of interest

The authors declare that they have no conflicts of interest with the contents of this article.
