# Supplementary material for: Quantification of NADH:ubiquinone oxidoreductase (complex I) content in biological samples
Source: J Biol Chem. 2021 Sep 17;297(4):101204. doi: 10.1016/j.jbc.2021.101204 (PMC8503622; doi:10.1016/j.jbc.2021.101204)
Supplement: Supplemental Table S1 [file mmc1.docx]

**Supplemental Table 1**. Most abundant mitochondrial proteins identified by LC-MS/MS in the flavin fluorescent gel bands F1 and F2 resolved by hrCNE from Fig. 1.

|  | **Protein name** | **M.w.**  **kDa** | **Access number (NCBI)** | **Band F1** | | | **Band F2** | | |
| --- | --- | --- | --- | --- | --- | --- | --- | --- | --- |
|  |  |  |  | Protein spectrum count^a^ | # Unique Peptides^b^ | Coverage % | Protein spectrum count^a^ | # Unique Peptides^b^ | Coverage % |
| 1 | 2-oxoglutarate dehydrogenase | 118.18 | Q60597-3 | 415 | 117 | 56 | 6 | 7 | 8 |
| 2 | Dihydrolipoyllysine- succinyltransferase | 48.99 | Q9D2G2 | 277 | 39 | 50 | 1 | 1 | 5 |
| 3 | OGDLH protein | 114.54 | B2RXT3_MOUSE | 234 | 60 | 54 | 3 | 1 | 5 |
| 4 | MICOS complex subunit Mic60 isoform 2 | 82.93 | Q8CAQ8-2 | 234 | 52 | 58 | 70 | 50 | 56 |
| 5 | ADP/ATP translocase 2 | 32.93 | ADT2_MOUSE | 90 | 28 | 63 | 32 | 20 | 46 |
| 6 | Dihydrolipoyl dehydrogenase | 54.27 | DLDH_MOUSE | 53 | 23 | 42 | 5 | 5 | 15 |
| 5 | NADH-ubiquinone oxidoreductase 75 kDa | 79.70 | Ndufs1 | 33 | 21 | 34 | 328 | 91 | 68 |
| 6 | ATP synthase subunit beta | 59.75 | Atp5f1b | 79 | 37 | 61 | 242 | 71 | 70 |
| 7 | NADH dehydrogenase flavoprotein 1 | 49.91 | Ndufv1 | 18 | 12 | 38 | 112 | 42 | 65 |
| 8 | NADH dehydrogenase FeS protein 2 | 52.63 | Ndufs2 | 20 | 12 | 26 | 107 | 43 | 65 |
| 9 | NADH dehydrogenase subunit 10 | 40.60 | Ndufa10 | 15 | 10 | 38 | 93 | 35 | 67 |
| 10 | NADH dehydrogenase subunit 9 | 42.12 | Ndufa9 | 16 | 11 | 34 | 71 | `41 | 60 |

^a^ Protein spectrum count was normalized by the total spectral count

^b^ The number of peptide sequences that are unique to a protein group
